# Association of neutrophil/high-density lipoprotein cholesterol ratio with the cardiovascular-kidney-metabolic syndrome and its cardiovascular mortality

**DOI:** 10.3389/fnut.2025.1594041

**Published:** 2025-06-04

**Authors:** Yaying Xu

**Affiliations:** Department of Endocrinology, The First Affiliated Hospital, and College of Clinical Medicine of Henan University of Science and Technology, Luoyang, China

**Keywords:** neutrophil/high-density lipoprotein cholesterol ratio, cardiovascular-kidney-metabolic syndrome, CVD mortality, NHANES, observational study

## Abstract

**Background:**

Cardiovascular-kidney-metabolic (CKM) syndrome is a novel multi-system disease defined by the American Heart Association (AHA). This study aims to investigate the associations between the neutrophil/high-density lipoprotein cholesterol ratio (NHR) and CKM syndrome and its association with cardiovascular disease (CVD) mortality.

**Methods:**

The data for this study were derived from the National Health and Nutrition Examination Survey (NHANES) conducted from 2005 to 2018 and the National Death Index (NDI) database. Weighted multivariate logistic regression and Cox regression were the primary analytical methods. restricted cubic spline (RCS) and subgroup analyses were also carried out.

**Results:**

A total of 13,165 participants were included in this study. The weighted mean of NHR was 3.14 ± 0.03. The prevalence rate of CKM syndrome was 17.36%. The results of the weighted logistic regression indicated that there was a positive association between NHR and CKM syndrome (OR_per SD_: 1.25, 95% CI: 1.10–1.42, *p* < 0.001). Among the quartile groups of NHR, the prevalence rate of CKM syndrome showed an increase (*p* for trend <0.0001). Among individuals with CKM syndrome, the results of the weighted Cox regression demonstrated that NHR also significantly predicted CVD mortality (HR_per SD_: 1.31, 95% CI: 1.14–1.50, *p* < 0.001). Among the quartile groups of NHR, the risk of CVD mortality also increased in a trend (*p* for trend = 0.01). Furthermore, RCS analysis showed a positive linear association between NHR and CKM syndrome (non-linear *p* = 0.075) and between NHR and CVD mortality (non-linear *p* = 0.300). The subgroup analysis suggested that the relationship between NHR and the outcomes was robust, and there was no significant interaction.

**Conclusion:**

Our study demonstrates that NHR is not only associated with an increased prevalence of CKM syndrome but also serves as an effective predictor for CVD mortality in patients with CKM syndrome. This provides new insights into the clinical identification and prognostic evaluation of CKM syndrome.

## Introduction

As a systemic disease, cardiovascular-kidney-metabolic (CKM) syndrome manifests as pathophysiological interactions among metabolic risk factors, chronic kidney disease (CKD), and the cardiovascular system. These interactions lead to the dysfunction of multiple organs and contribute to a high incidence of adverse cardiovascular outcomes (1). When metabolic abnormalities, cardiovascular disease (CVD), and CKD are intricately interconnected and simultaneously present in an individual, it results in a significantly worse prognosis. Notably, these diseases may share common pathogenic mechanisms. That is to say, the progression of one of these diseases is highly likely to exacerbate the others, thereby further deteriorating the overall health status (2). CKM syndrome can be classified into five stages ranging from 0 to 4 according to the severity of the condition. Among them, stages 3 and 4 are considered advanced stages. Relevant data indicate that in the United States, nearly 90% of adults meet the diagnostic criteria for stage 1 of CKM syndrome, while approximately 15% of the population meet the diagnostic criteria for the advanced stages (stages 3–4) (3). Given the high prevalence of CKM syndrome, both public health professionals and frontline clinical workers should pay more attention to it. From the perspective of disease management, accurate early identification and continuous monitoring of CKM syndrome are crucial prerequisites for effective management.

In the pathogenesis of CVD, CKD, and metabolic disorders, inflammation is a key factor shared by all three. Chronic, low-level inflammation plays a crucial and non-negligible role in the pathophysiological processes involved in metabolic abnormalities, CVD, and kidney damage. For instance, in the case of obesity, especially when there is a significant accumulation of visceral fat, macrophages within adipose tissues are activated, leading to the release of pro-inflammatory cytokines such as TNF-α and IL-6, as well as chemokines. This triggers a systemic inflammatory response. Such an inflammatory response exerts an inhibitory effect along the insulin signaling pathway, exacerbating the disorder of glucose and lipid metabolism (4). At the same time, inflammatory mediators can promote the formation of atherosclerotic plaques by causing endothelial dysfunction and oxidative stress, accelerating the deterioration of coronary artery disease and heart failure (5, 6). When focusing on the field of the kidneys, inflammation can drive the infiltration of immune cells into the glomeruli, damage podocytes, and subsequently lead to tubulointerstitial fibrosis. Eventually, this results in a decrease in the glomerular filtration rate (GFR) and the emergence of proteinuria (7, 8). Notably, recent research indicates that the Systemic Immune-Inflammation Index (SII) is highly likely to become a powerful tool for predicting CKM syndrome, providing valuable reference indicators for research and clinical practice in this field (9).

The neutrophil/high-density lipoprotein cholesterol ratio (NHR), a comprehensive indicator integrating inflammatory cells and lipid metabolism, has been proven to be associated with a higher risk of CKD (10), the risk of death in the general population (11), and an increased prevalence of metabolic syndrome (MetS) (12). CKM syndrome is a systemic disease resulting from the pathophysiological interactions among obesity, type 2 diabetes mellitus (T2DM), CKD, and CVD. However, the relationship between CKM syndrome and NHR remains unclear.

This study aims to explore the relationship between NHR and the prevalence of CKM syndrome and the CVD mortality rate among the adult population in the United States. It is intended to provide evidence for the early identification and long-term prognosis of CKM syndrome.

## Methods

### Participants

**Figure 1:**
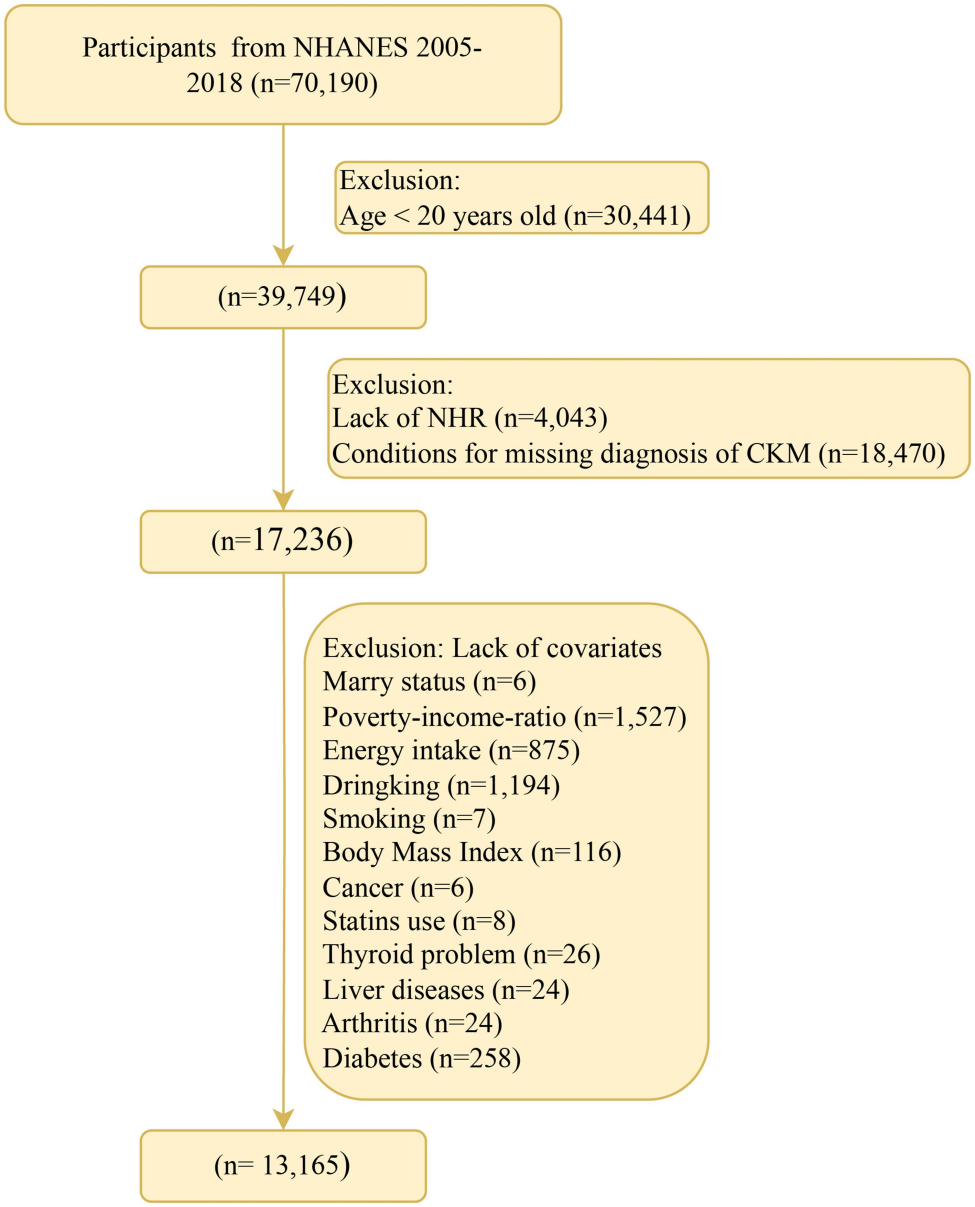
Flow chart of participant recruitment. NHR, neutrophil/high-density lipoprotein cholesterol ratio; NHANES, National Health and Nutrition Examination Survey; CKM, cardiovascular-kidney-metabolic syndrome.

The National Health and Nutrition Examination Survey (NHANES) in the United States is a continuous national cross-sectional research project conducted every 2 years for the American population. Through personal interviews, filling out standardized questionnaires, and physical examinations, this survey collects participants’ basic demographic data, socioeconomic background information, and data related to health and nutritional status. The Ethics Review Committee of the National Center for Health Statistics in the United States has approved all NHANES research plans, and each individual participating in the survey has signed a written informed consent form. The data for this study are sourced from seven non-overlapping cycles of NHANES from 2005 to 2018, and the specific inclusion and exclusion steps can be referred to in Figure 1. During this time period, a total of 70,190 people participated in the survey. Among them, 30,441 individuals were under 20 years of age; 4,043 people had missing NHR data; 18,470 people did not have the necessary information for diagnosing CKM syndrome; and another 4,071 people were excluded in the subsequent step-by-step screening process due to the lack of covariates. Considering all the above situations, a total of 13,165 participants finally met all the inclusion requirements of this study.

### NHR

The count of neutrophils is determined by performing a complete blood cell count using an automated blood analysis system, and the final result is presented in the form of 10 to the power of 3 cells per microliter, that is, 10^3^ cells/μL. As for determining the high-density lipoprotein cholesterol (HDL-C) level, an automatic detection device is used to test and evaluate the venous blood samples collected after the subjects have fasted for 8 h. NHR is the quotient obtained by dividing the neutrophil count (10^3^ cells/μL) by the HDL-C level (mmol/L) (11).

### Diagnosis of CKM syndrome

The concept of CKM syndrome was recently proposed by the American Heart Association (AHA) and Aggarwal et al. (3, 13). This syndrome is subdivided into five different stages, ranging from stage 0 to stage 4, according to the degree of disease progression. Specifically, stage 0 is defined as having a normal body mass index (BMI) and waist circumference, and these participants will not be classified into a higher stage category. When participants exhibit an increase in BMI value, or an enlargement of waist size, or meet the corresponding characteristics of prediabetes, they are classified as stage 1. Participants in stage 2 have metabolic-related risk factors or are at a moderately high risk of developing CKD. Stage 3 is defined as participants having a high risk of CKD or being predicted to have a high risk of developing CVD according to the 10-year CVD prediction model. If participants already have CVD, they belong to stage 4 (14). It is particularly important to note that the determination of the risk of developing CKD is based on the standards set by Kidney Disease Improving Global Outcomes (KDIGO) (15); while the risk of developing CVD is estimated by using the Prediction of Cardiovascular Events (PREVENT) equation (16). More detailed information can be found in Supplementary Table 1. According to previous relevant research conclusions, stages 3–4 are generally classified as CKM syndrome (17).

### CVD mortality

The National Death Index (NDI) in the United States records the death status. By matching each participant of NHANES with the NDI database, the survival status of the individuals in this study was determined. Specifically, if the matching was successful, it indicated that the individual had been confirmed to be deceased, and the date of death was recorded. If the matching was unsuccessful, it meant that the individual was still alive, and the follow-up end date for such individuals was recorded as 31 December 2019 (18, 19). The follow-up duration was calculated as the difference between the date of death or the follow-up end date and the recruitment date. The specific death status due to CVD was determined according to the guidelines of the International Classification of Diseases, 10th edition (ICD-10), which includes: (1) heart diseases (ICD-10 codes I00–I09, I11, I13, I20–I51); and (2) cerebrovascular diseases (ICD-10 codes I60–I69).

### Covariates

When conducting this study, we extensively reviewed a large number of relevant previous works of literature and determined the following confounding factors in close combination with the unique characteristics of the NHANES database (20, 21). On the one hand, in the variable collection stage, we focused on the demographic and socioeconomic categories. The specific variables covered include a categorical variable for individual gender; a continuous variable for individual age that accurately reflects the age changes; a racial variable subdivided into five categories according to different ethnic characteristics; an educational background variable divided into two categories, namely a college degree or above and other levels, based on the differences in educational attainment; a marital status variable classified into three categories according to the actual marital situation; and a poverty-income ratio (PIR) variable, also divided into three categories, constructed according to the relationship between poverty and income. On the other hand, we comprehensively collected variables related to lifestyle and physical indicators. For example, a smoking status variable subdivided into three categories according to the different degrees and frequencies of smoking behavior; a three-category variable divided according to drinking habits; a physical activity variable classified into three categories, namely less than 700 min of weekly physical activity, between 700 and 2,400 min, and more than 2,400 min, based on the length of weekly physical activity time; a continuous variable: total dietary energy intake, which can intuitively show the overall level of an individual’s dietary energy intake; and a body mass index (BMI), also a continuous variable, used to measure the proportional relationship between an individual’s body mass and height. Moreover, during the research process, we also fully considered various characteristic factors closely related to health. These include a binary variable to determine whether an individual has cancer; a binary variable to judge whether there are thyroid problems; a binary variable to identify whether an individual has arthritis; a binary variable to examine whether there are liver diseases; a diabetes status variable divided into three categories, namely no, yes, and prediabetes, to define the state of diabetes; a binary variable to confirm whether an individual has hypertension; a binary variable to consider whether statin drugs are used; and a concentration variable reflecting the level of total cholesterol (TC) in the serum.

### Statistical analysis

When describing the characteristics of the participants, for the comparison between continuous variables, the Wilcoxon rank-sum test was used, and the corresponding results were presented in the form of mean values and standard errors (SEs). For categorical variables, the chi-square test was applied, and they were expressed using the number of cases (*n*) and the percentage (%). Considering compliance with statistical norms and facilitating subsequent interpretation, before being incorporated into the multivariate logistic regression model or the multivariate COX proportional hazards model, NHR was standardized and transformed into a *Z*-score. Subsequently, the associations between exposure factors and outcomes were evaluated, specifically estimating the odds ratios (ORs), hazard ratios (HRs), and 95% confidence intervals (CIs). Several models were constructed during the research process. Model 0 made no adjustments to the covariates, aiming to provide a basic reference. Model 1 included and adjusted for covariates such as gender, age, race, educational attainment, marital status, and family economic status, initially controlling some interfering factors at the level of individual basic characteristics. Model 2 went further by including factors such as alcohol consumption, smoking, exercise level, dietary energy intake, and BMI in the adjustment scope. Model 3, based on the above, continued to adjust for factors related to comorbidities, medication history, and serum TC level, covering hypertension, diabetes, thyroid diseases, liver diseases, arthritis, tumors, history of statin use, and serum TC level, striving to exclude the influence of various potential confounding factors as much as possible. To ensure the reliability of the models, the variance inflation factor (VIF) was used to check for collinearity issues. In this study, all VIF values were less than 10, effectively avoiding the misguidance of collinearity on the results. Meanwhile, Schoenfeld residuals were used to test the proportional hazards assumption. The test showed that this study fully conformed to this premise assumption, ensuring the applicability of the COX proportional hazards model. In addition, subgroup analysis was carried out, and the likelihood ratio test was used to explore the interaction between covariates and NHR, aiming to uncover potential variable association patterns. Finally, restricted cubic splines (RCS) were employed to evaluate the dose-response relationship between the continuously changing NHR and the risks of CKM syndrome and CVD mortality, delicately depicting the dynamic associations between variables.

It is worth noting that all statistical procedures involved in this study were completed relying on R software version 4.3.3 (R Foundation for Statistical Computing). In the current study, a two-tailed *p*-value less than 0.05 was determined to be statistically significant and used as the criterion for judging the significance of the results.

## Results

### Population characteristics

According to the research design, the participants were grouped based on whether they had CKM syndrome, and the corresponding baseline characteristics are presented in Table 1. Under the strict screening of the inclusion criteria, this study involved a total of 13,165 participants, among whom 2,285 individuals were diagnosed with CKM syndrome, accounting for a weighted proportion of 17.36%. After performing a weighted average on the age data of all participants, the average age was found to be 47.68 (±0.28) years, and there were 6,525 female participants, accounting for 49.97% of the total number of participants. Compared with the group without CKM syndrome, the NHR of the participants with CKM syndrome was significantly higher. In terms of demographic characteristics, among the patients with CKM syndrome, there was a higher proportion of males in terms of gender distribution; they tended to be in the older age group in terms of age structure; the proportion of non-Hispanic White people increased in terms of racial categories; those with lower educational attainment were more common in the field of education; the proportion of divorced or unmarried individuals was higher in the category of marital status; and the proportion of people living in poverty was larger from the perspective of economic conditions. In terms of lifestyle, patients with CKM syndrome were more likely to have a history of smoking and drinking, a lower level of dietary energy intake, and relatively less daily physical activity. As for comorbidities, the incidence of obesity was higher in this group. At the same time, the prevalence rates of diseases such as cancer, thyroid problems, liver diseases, arthritis, diabetes, and hypertension were all significantly higher than those in the group without CKM syndrome. It is worth noting that in terms of medication use and serum indicators, participants with CKM syndrome had a higher probability of using statin drugs, while their serum TC levels were relatively lower.

**Table 1:** Baseline characteristics based on whether participants have CKM syndrome for cross-sectional study.

Characteristics	Total (*n* = 13,165)	Without CKM (*n* = 10,880)	With CKM (*n* = 2,285)	*p*-value
NHR, mean (SE)	3.14 (0.03)	3.06 (0.03)	3.64 (0.06)	<0.0001
Age, mean (SE)	47.68 (0.28)	44.76 (0.29)	67.54 (0.40)	<0.0001
Sex, *n* (%)				<0.0001
Female	6,525 (49.97)	5,595 (50.95)	930 (43.37)	
Male	6,640 (50.03)	5,285 (49.05)	1,355 (56.63)	
Race, *n* (%)				<0.0001
Mexican American	2,021 (7.96)	1,800 (8.51)	221 (4.24)	
Non-Hispanic Black	2,619 (9.86)	2,149 (9.75)	470 (10.59)	
Non-Hispanic White	6,040 (70.56)	4,728 (69.65)	1,312 (76.75)	
Other Hispanic	1,207 (4.96)	1,031 (5.15)	176 (3.66)	
Other race—including multi-racial	1,278 (6.65)	1,172 (6.93)	106 (4.76)	
Educational level, *n* (%)				<0.0001
No college	6,118 (38.48)	4,784 (36.44)	1,334 (52.29)	
College or equivalent	7,047 (61.52)	6,096 (63.56)	951 (47.71)	
Marital status, *n* (%)				<0.0001
No married	2,315 (17.25)	2,197 (19.15)	118 (4.35)	
Divorced or separated or widowed	2,897 (18.19)	2,044 (16.00)	853 (33.03)	
Already married or cohabitation	7,953 (64.56)	6,639 (64.84)	1,314 (62.63)	
PIR, *n* (%)				<0.0001
<1.3	3,986 (20.11)	3,188 (19.19)	798 (26.34)	
1.3–3.5	5,056 (36.25)	4,062 (35.11)	994 (44.00)	
>3.5	4,123 (43.64)	3,630 (45.70)	493 (29.65)	
Drinking status, *n* (%)				<0.0001
Never drink	1,730 (10.29)	1,373 (9.79)	357 (13.64)	
Former drinker	2,180 (13.51)	1,479 (11.34)	701 (28.26)	
Current drinker	9,255 (76.20)	8,028 (78.87)	1,227 (58.10)	
Smoking status, *n* (%)				<0.0001
Never smoked	7,137 (54.23)	6,188 (56.24)	949 (40.58)	
Former smoker	3,302 (25.77)	2,422 (23.88)	880 (38.62)	
Current smoker	2,726 (19.99)	2,270 (19.88)	456 (20.80)	
Physical activity (MET, minutes/week, *n* (%)				<0.0001
<700	2,581 (19.85)	2,139 (19.82)	442 (20.05)	
700–2,400	2,995 (24.60)	2,556 (25.28)	439 (20.01)	
≥2,400	4,329 (35.13)	3,880 (37.04)	449 (22.15)	
Not report	3,260 (20.42)	2,305 (17.86)	955 (37.79)	
Energy intake (kcal/day), mean (SE)	2,188.64 (11.26)	2,230.23 (11.69)	1,906.46 (30.94)	<0.0001
BMI, mean (SE)	29.03 (0.10)	28.87 (0.11)	30.10 (0.21)	<0.0001
Cancer, *n* (%)				<0.0001
No	11,942 (90.42)	10,155 (92.45)	1,787 (76.63)	
Yes	1,223 (9.58)	725 (7.55)	498 (23.37)	
Thyroid problem, *n* (%)				<0.0001
No	11,799 (89.18)	9,914 (90.42)	1,885 (80.74)	
Yes	1,366 (10.82)	966 (9.58)	400 (19.26)	
Liver problem, *n* (%)				<0.0001
No	12,640 (96.21)	10,498 (96.65)	2,142 (93.21)	
Yes	525 (3.79)	382 (3.35)	143 (6.79)	
Arthritis, *n* (%)				<0.0001
No	9,493 (73.34)	8,410 (77.25)	1,083 (46.79)	
Yes	3,672 (26.66)	2,470 (22.75)	1,202 (53.21)	
DM, *n* (%)				<0.0001
DM	2,761 (15.87)	1,638 (11.76)	1,123 (43.76)	
IFG	1,285 (9.99)	1,055 (9.77)	230 (11.51)	
IGT	1,100 (7.51)	889 (7.32)	211 (8.77)	
No	8,019 (66.63)	7,298 (71.15)	721 (35.97)	
Hypertension, *n* (%)				<0.0001
No	7,544 (61.78)	7,047 (67.28)	497 (24.44)	
Yes	5,621 (38.22)	3,833 (32.72)	1,788 (75.56)	
Statins, *n* (%)				<0.0001
No	10,604 (82.03)	9,446 (87.27)	1,158 (46.47)	
Yes	2,561 (17.97)	1,434 (12.73)	1,127 (53.53)	
Total cholesterol (mmol/L), mean (SE)	5.00 (0.02)	5.04 (0.02)	4.73 (0.03)	<0.0001

CKM, cardiovascular-kidney-metabolic syndrome; NHR, neutrophil/high-density lipoprotein cholesterol ratio; SE, standard error; PIR, poverty-to-income ratio; MET, metabolic equivalent; DM, diabetes mellitus; IFG, impaired fasting glycemia; IGT, impaired glucose tolerance.

In the survival analysis, among the initial 2,285 patients with CKM syndrome, 564 individuals who died from non-CVD causes were excluded, and 1,721 participants were included. Among them, 306 individuals died due to CVD-specific causes. Compared with the survivor group, the NHR of the deceased group tended to be higher (*p* = 0.09). The deceased group was more likely to be older, divorced, less wealthy, have a history of drinking, have never smoked, be less physically active, have a low dietary energy intake, and have a low BMI. In addition, the deceased group was more likely to have diabetes and hypertension (Table 2).

**Table 2:** Baseline characteristics of the participants with CKM syndrome in survival analysis.

Characteristics	Total (*n* = 1721)	Survivals (*n* = 1,415)	CVD mortality (*n* = 306)	*p*-value
Follow-up duration (years), mean (SE)	6.95 (0.15)	7.21 (0.17)	5.43 (0.19)	<0.0001
NHR, mean (SE)	3.62 (0.06)	3.58 (0.07)	3.82 (0.12)	0.09
Age, mean (SE)	65.87 (0.42)	64.39 (0.44)	74.41 (0.63)	<0.0001
Sex, *n* (%)				0.89
Female	717 (43.04)	607 (43.11)	110 (42.62)	
Male	1,004 (56.96)	808 (56.89)	196 (57.38)	
Race, *n* (%)				0.05
Mexican American	177 (4.50)	160 (4.86)	17 (2.47)	
Non-Hispanic Black	385 (11.35)	328 (11.62)	57 (9.79)	
Non-Hispanic White	923 (74.93)	716 (73.73)	207 (81.84)	
Other Hispanic	145 (4.08)	132 (4.41)	13 (2.15)	
Other race—including multi-racial	91 (5.14)	79 (5.39)	12 (3.75)	
Educational level, *n* (%)				0.14
No college	955 (49.27)	776 (48.46)	179 (53.98)	
College or equivalent	766 (50.73)	639 (51.54)	127 (46.02)	
Marital status, *n* (%)				0.003
No married	100 (4.82)	85 (4.82)	15 (4.80)	
Divorced or separated or widowed	602 (30.66)	473 (28.80)	129 (41.44)	
Already married or cohabitation	1,019 (64.52)	857 (66.38)	162 (53.76)	
PIR, *n* (%)				<0.001
<1.3	582 (24.85)	483 (24.68)	99 (25.79)	
1.3–3.5	738 (42.24)	582 (40.29)	156 (53.48)	
>3.5	401 (32.91)	350 (35.02)	51 (20.73)	
Drinking status, *n* (%)				<0.0001
Never drink	259 (12.20)	206 (11.31)	53 (17.38)	
Former drinker	476 (25.97)	354 (23.93)	122 (37.75)	
Current drinker	986 (61.82)	855 (64.76)	131 (44.87)	
Smoking status, *n* (%)				<0.001
Never smoked	743 (41.82)	585 (39.99)	158 (52.42)	
Former smoker	622 (36.68)	509 (36.59)	113 (37.24)	
Current smoker	356 (21.49)	321 (23.42)	35 (10.34)	
Physical activity (MET, minutes/week, *n* (%)				<0.0001
<700	327 (20.02)	273 (20.31)	54 (18.31)	
700–2,400	350 (20.95)	289 (20.79)	61 (21.89)	
≥2,400	395 (25.77)	358 (28.40)	37 (10.57)	
Not report	649 (33.26)	495 (30.49)	154 (49.23)	
Energy intake (kcal/day), mean (SE)	1955.26 (36.81)	1982.97 (39.81)	1795.26 (60.02)	0.005
BMI, mean (SE)	30.55 (0.24)	30.78 (0.27)	29.20 (0.44)	0.003
Cancer, *n* (%)				0.05
No	1,385 (78.96)	1,154 (79.96)	231 (73.22)	
Yes	336 (21.04)	261 (20.04)	75 (26.78)	
Thyroid problem, *n* (%)				0.77
No	1,418 (81.31)	1,159 (81.18)	259 (82.03)	
Yes	303 (18.69)	256 (18.82)	47 (17.97)	
Liver problem, n (%)				0.72
No	1,612 (93.07)	1,328 (93.17)	284 (92.51)	
Yes	109 (6.93)	87 (6.83)	22 (7.49)	
Arthritis, *n* (%)				0.87
No	824 (47.61)	681 (47.71)	143 (47.01)	
Yes	897 (52.39)	734 (52.29)	163 (52.99)	
DM, *n* (%)				0.01
DM	825 (42.00)	676 (41.32)	149 (45.92)	
IFG	167 (11.64)	144 (12.13)	23 (8.81)	
IGT	145 (7.72)	104 (6.76)	41 (13.29)	
No	584 (38.64)	491 (39.79)	93 (31.98)	
Hypertension, *n* (%)				0.003
No	379 (25.22)	323 (26.78)	56 (16.26)	
Yes	1,342 (74.78)	1,092 (73.22)	250 (83.74)	
Statins, *n* (%)				0.6
No	865 (45.64)	714 (45.35)	151 (47.37)	
Yes	856 (54.36)	701 (54.65)	155 (52.63)	
Total cholesterol (mmol/L), mean (SE)	4.73 (0.04)	4.74 (0.04)	4.67 (0.07)	0.39

CKM, cardiovascular-kidney-metabolic syndrome; NHR, neutrophil/high-density lipoprotein cholesterol ratio; SE, standard error; PIR, poverty-to-income ratio; MET, metabolic equivalent; DM, diabetes mellitus; IFG, impaired fasting glycemia; IGT, impaired glucose tolerance.

### Estimation of the association of NHR with CKM syndrome

Table 3 shows the relationship between NHR and CKM syndrome analyzed through the weighted logistic regression model. In the unadjusted model (Model 0), an increase of one standard deviation (SD) in NHR was associated with a 43% increase in the prevalence of CKM syndrome (OR: 1.43, 95% CI: 1.34–1.53, *p* < 0.0001). In the model with adjusted demographic characteristics (Model 1), the association between NHR and CKM syndrome was stronger (OR: 1.70, 95% CI: 1.53–1.90, *p* < 0.0001). In the model with further adjustment of lifestyle factors (Model 2), the association weakened somewhat (OR: 1.43, 95% CI: 1.27–1.61, *p* < 0.0001). Even in the fully adjusted Model 3, this significant positive association still existed. For every increase of one SD in NHR, the prevalence of CKM syndrome increased by 25% (OR: 1.25, 95% CI: 1.10–1.42, *p* < 0.001). In the model with NHR categorized into four groups, compared with the Quartile 1 group, in any model, the prevalence of CKM syndrome in both the Quartile 3 group and the Quartile 4 group was significantly higher than that in the Quartile 1 group. In the fully adjusted model, the prevalence of CKM syndrome in the Quartile 3 group (OR: 1.30, 95% CI: 1.00–1.69, *p* = 0.05) and the Quartile 4 group (OR: 1.74, 95% CI: 1.34–2.26, *p* < 0.0001) increased by 30 and 74%, respectively.

**Table 3:** OR estimates for the association between NHR and CKM syndrome.

		Model 0		Model 1		Model 2		Model 3	
		OR (95% CI)	*p-*value	OR (95% CI)	*p-*value	OR (95% CI)	*p-*value	OR (95% CI)	*p-*value
CKM ~ NHR	Per SD	1.43 (1.34, 1.53)	<0.0001	1.70 (1.53, 1.90)	<0.0001	1.43 (1.27, 1.61)	<0.0001	1.25 (1.10, 1.42)	<0.001
	Quartile 1	Reference		Reference		Reference		Reference	
	Quartile 2	1.44 (1.18, 1.75)	<0.001	1.60 (1.26, 2.02)	<0.001	1.44 (1.13, 1.84)	0.004	1.20 (0.94, 1.53)	0.14
	Quartile 3	1.65 (1.39, 1.96)	<0.0001	2.01 (1.57, 2.58)	<0.0001	1.61 (1.25, 2.07)	<0.001	1.30 (1.00, 1.69)	0.05
	Quartile 4	2.47 (2.07, 2.96)	<0.0001	3.53 (2.78, 4.50)	<0.0001	2.43 (1.87, 3.15)	<0.0001	1.74 (1.34, 2.26)	<0.0001
	*P* for trend		<0.0001		<0.0001		<0.0001		<0.0001

Model 0: Crude model. Model 1: Adjusted for age, sex, race, marital status, education, and poverty-income ratio. Model 2: Additionally adjusted for drinking, smoking, total energy intake, weekly physical activity level, and body mass index. Model 3: Further adjusted for diabetes, cancer, hypertension, liver diseases, thyroid diseases, arthritis, statins, and the levels of serum total cholesterol. CKM, cardiovascular-kidney-metabolic syndrome; NHR, neutrophil/high-density lipoprotein cholesterol ratio; SD, standard deviation; OR, odds ratio; CI, confidence interval.

**Figure 2:**
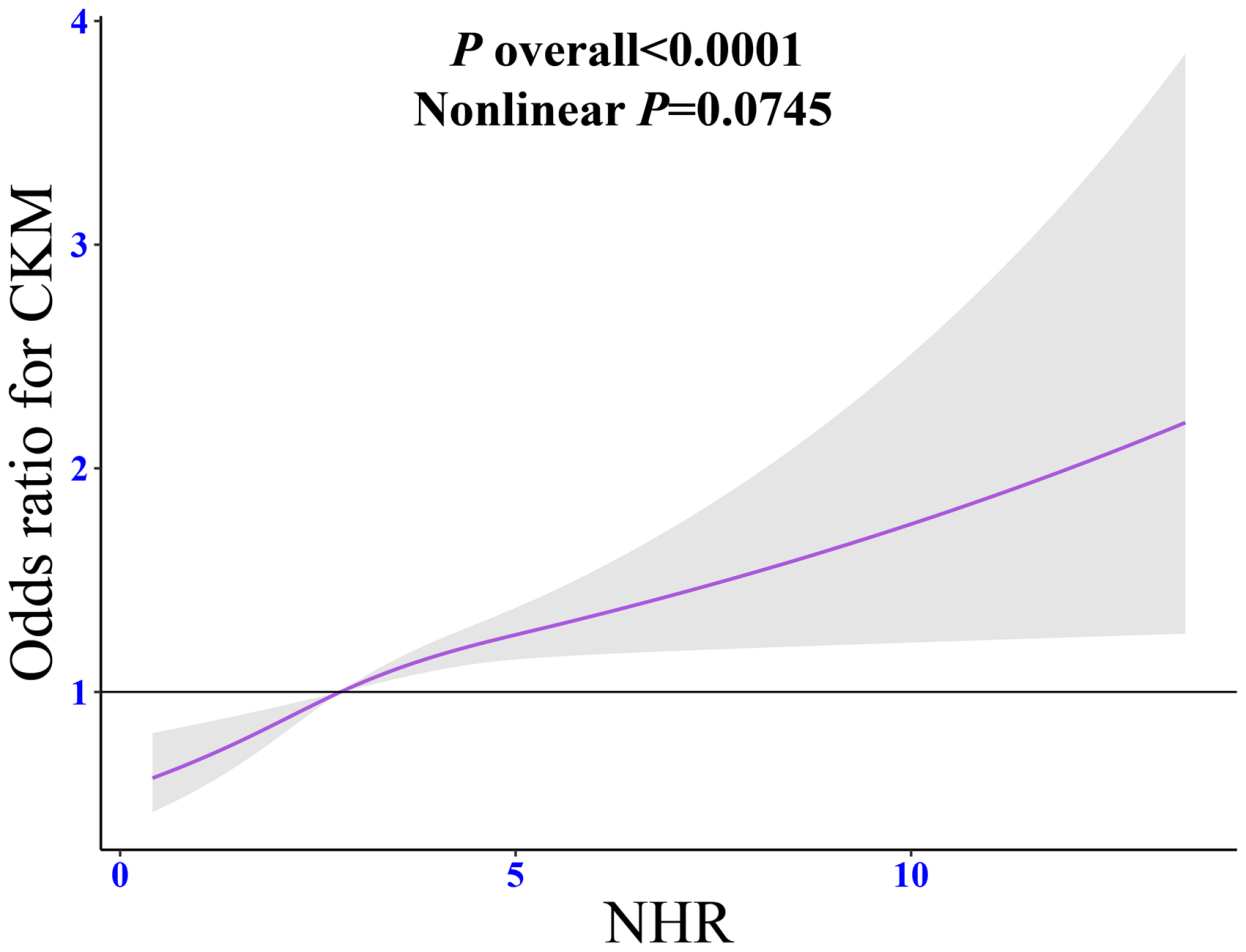
Dose-response relationship between NHR and CKM syndrome. The adjusted restricted cubic spline model was adjusted for age, sex, race, marital status, education, poverty-to-income ratio, drinking, smoking, total energy intake, weekly physical activity level, body mass index, diabetes, cancer, hypertension, liver diseases, thyroid diseases, arthritis, statins, and the levels of serum total cholesterol. NHR, neutrophil/high-density lipoprotein cholesterol ratio; CKM, cardiovascular-kidney-metabolic syndrome; OR, odds ratio; CI, confidence interval.

RCS regression model with adjustment for all confounding factors showed a linear dose relationship between NHR and CKM syndrome (non-linear *p* = 0.075). As the NHR level increased, the prevalence of CKM syndrome increased steadily (Figure 2).

### Estimation of the association between NHR and CVD mortality among patients with CKM syndrome

Table 4 shows the relationship between NHR and CVD mortality analyzed through the weighted Cox regression model. In the unadjusted model (Model 0), NHR was not associated with the risk of CVD mortality (HR: 1.10, 95% CI: 0.99–1.23, *p* = 0.07). In the model with adjusted demographic characteristics (Model 1), NHR increased the risk of CVD mortality (HR: 1.28, 95% CI: 1.13–1.46, *p* < 0.001). In the model with further adjustment of lifestyle factors (Model 2), the association between them was strengthened (HR: 1.31, 95% CI: 1.15–1.50, *p* < 0.0001). Even in the fully adjusted Model 3, this significant positive association still existed. For every increase per SD in NHR, the risk of CVD mortality increased by 31% (HR: 1.31, 95% CI: 1.14–1.50, *p* < 0.001). In the model with NHR categorized into four groups, from Model 0 to Model 3, compared with the Quartile 1 group, the risk of CVD mortality in the Quartile 4 group was significantly higher. After full adjustment (Model 3), the risk of CVD mortality in the Quartile 4 group increased by 70% compared with the Quartile 1 group (HR: 1.70, 95% CI: 1.13–2.57, *p* = 0.01, *p*_trend_ = 0.01).

**Table 4:** HR estimates for the association between NHR and CVD mortality in patients with CKM syndrome.

		Model 0		Model 1		Model 2		Model 3	
		HR (95% CI)	*p-*value	HR (95% CI)	*p-*value	HR (95% CI)	*p-*value	HR (95% CI)	*p-*value
CVD mortality ~ NHR	Continuous	1.10 (0.99, 1.23)	0.07	1.28 (1.13, 1.46)	<0.001	1.31 (1.15, 1.50)	<0.0001	1.31 (1.14, 1.50)	<0.001
	Quartile 1	Reference		Reference		Reference		Reference	
	Quartile 2	0.89 (0.59, 1.33)	0.56	0.94 (0.64, 1.36)	0.73	0.91 (0.63, 1.29)	0.59	0.90 (0.63, 1.29)	0.58
	Quartile 3	1.01 (0.66, 1.54)	0.98	1.06 (0.71, 1.58)	0.78	1.08 (0.72, 1.61)	0.72	1.05 (0.67, 1.66)	0.83
	Quartile 4	1.33 (0.92, 1.92)	0.13	1.62 (1.13, 2.33)	0.01	1.78 (1.21, 2.60)	0.003	1.70 (1.13, 2.57)	0.01
	*p* for trend		0.07		0.01		0.003		0.01

Model 0: Crude model. Model 1: Adjusted for age, sex, race, marital status, education, and poverty-income ratio. Model 2: Additionally adjusted for drinking, smoking, total energy intake, weekly physical activity level, and body mass index. Model 3: Further adjusted for diabetes, cancer, hypertension, liver diseases, thyroid diseases, arthritis, statins, and the levels of serum total cholesterol. CKM, cardiovascular-kidney-metabolic syndrome; NHR, neutrophil/high-density lipoprotein cholesterol ratio; SD, standard deviation; HR, hazard ratio; CI, confidence interval.

**Figure 3:**
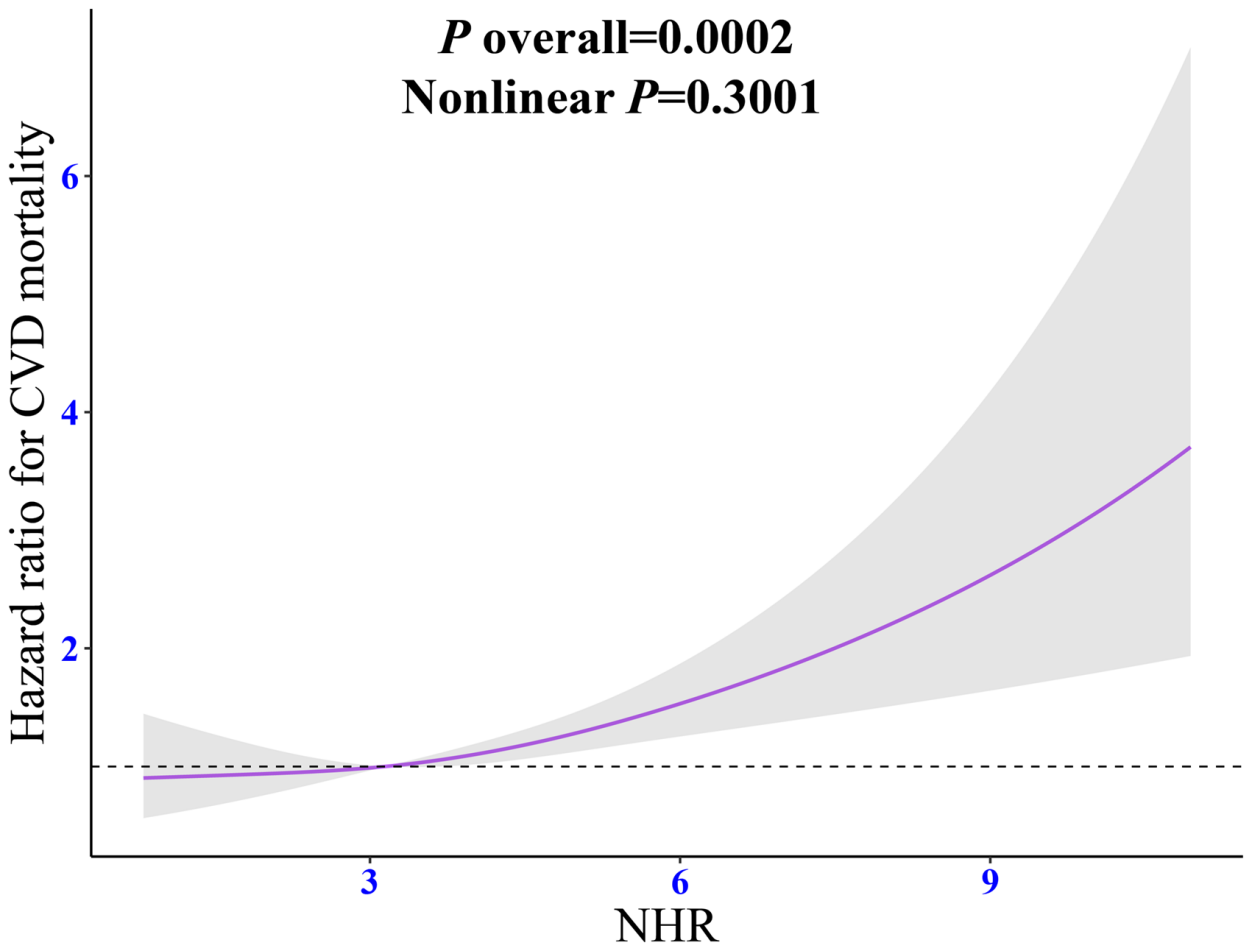
Dose-response relationship between NHR and CVD mortality among individuals with CKM syndrome. The adjusted restricted cubic spline model was adjusted for age, sex, race, marital status, education, poverty-to-income ratio, drinking, smoking, total energy intake, weekly physical activity level, body mass index, diabetes, cancer, hypertension, liver diseases, thyroid diseases, arthritis, statins, and the levels of serum total cholesterol. NHR, neutrophil/high-density lipoprotein cholesterol ratio; CKM, cardiovascular-kidney-metabolic syndrome; HR, hazard ratio; CI, confidence interval.

RCS regression model with adjustment for all confounding factors showed a linear dose relationship between NHR and the risk of CVD mortality (non-linear *p* = 0.300). As the NHR level increased, the risk of CVD mortality increased steadily (Figure 3).

### Subgroup analysis

**Figure 4:**
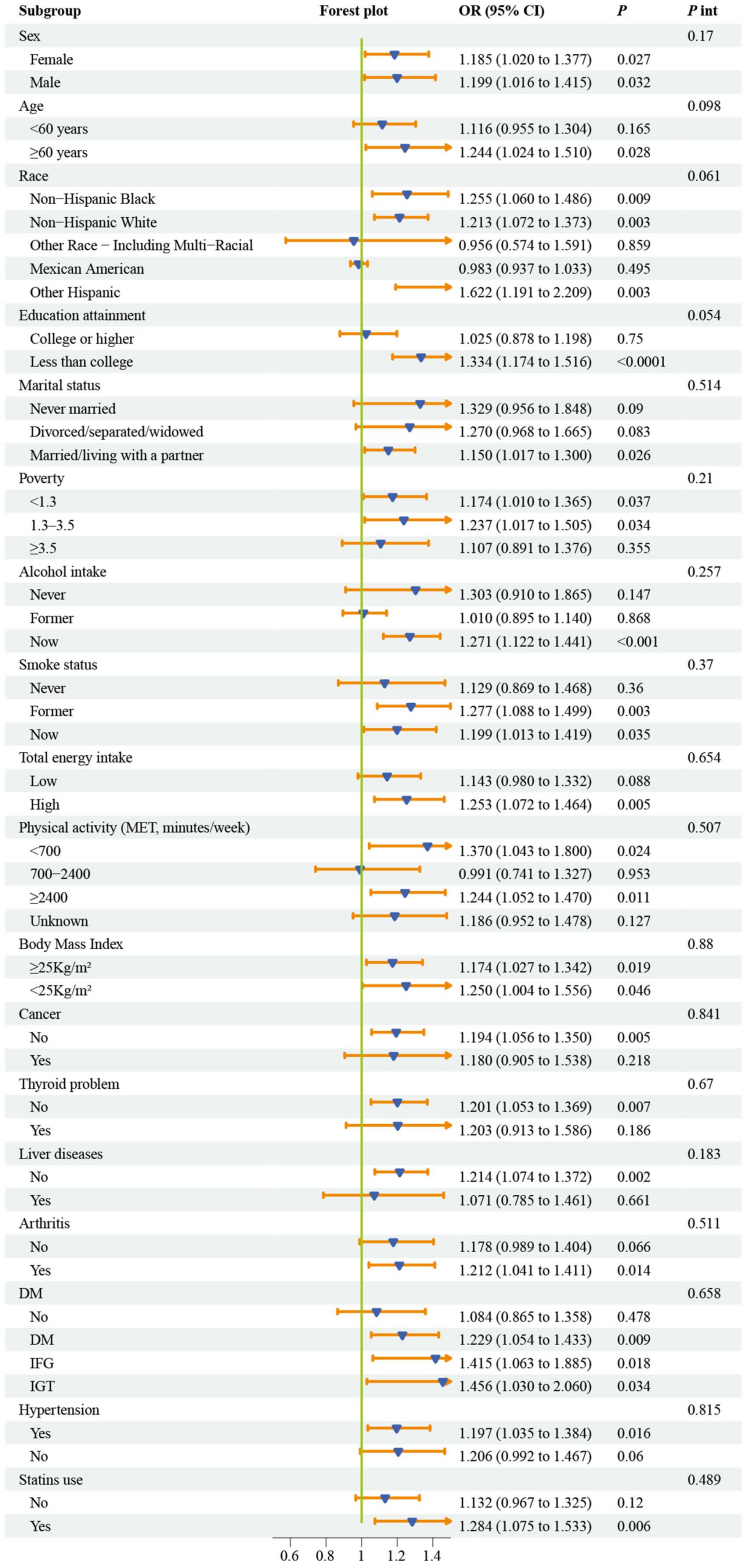
Subgroup analyses for the association between NHR and CKM syndrome. Models were adjusted for age, sex, race, marital status, education, poverty-to-income ratio, drinking, smoking, total energy intake, weekly physical activity level, body mass index, diabetes, cancer, hypertension, liver diseases, thyroid diseases, arthritis, statins, and the levels of serum total cholesterol. CKM, cardiovascular-kidney-metabolic syndrome; OR, odds ratio; NHR, neutrophil/high-density lipoprotein cholesterol ratio; PIR, poverty-to-income ratio; MET, metabolic equivalent; DM, diabetes mellitus; IFG, impaired fasting glycemia; IGT, impaired glucose tolerance; OR, odds ratio; CI, confidence interval.

Figure 4 reveals the differences in the relationship between NHR and CKM syndrome among different subgroups. In the fully adjusted model, excluding the stratification factors, the association between NHR and CKM syndrome was robust (*p*_interaction_ >0.05).

**Figure 5:**
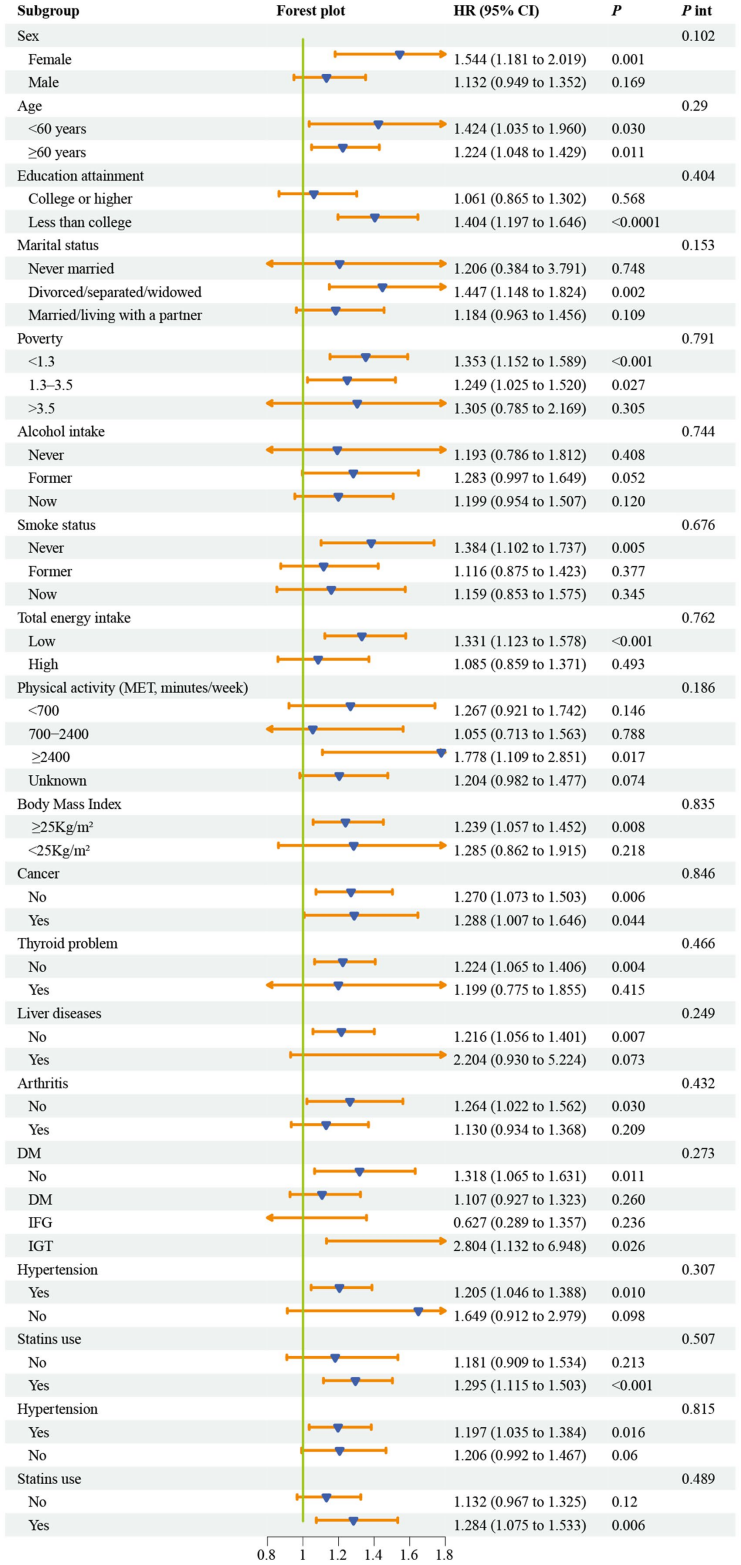
Subgroup analyses for the association between NHR and the risk of CVD mortality among individuals with CKM syndrome. Models were adjusted for age, sex, race, marital status, education, poverty-to-income ratio, drinking, smoking, total energy intake, weekly physical activity level, body mass index, diabetes, cancer, hypertension, liver diseases, thyroid diseases, arthritis, statins, and the levels of serum total cholesterol. CKM, cardiovascular-kidney-metabolic syndrome; NHR, neutrophil/high-density lipoprotein cholesterol ratio; MET, metabolic equivalent; DM, diabetes mellitus; IFG, impaired fasting glycemia; IGT, impaired glucose tolerance; HR, hazard ratio; CI, confidence interval.

Figure 5 shows the results of the association between NHR and CVD mortality among different subgroups. After fully adjusting for all confounding factors except the stratification variables, the association between NHR and CVD mortality was also robust (*p*_interaction_ >0.05).

## Discussion

In this large-scale cross-sectional study based on NHANES, the relationship between NHR and CKM syndrome was comprehensively investigated. Specifically, this study observed that an increase in NHR was associated with an elevated prevalence of CKM syndrome, and this association was linear and remained stable across different populations. In addition, this study also linked the NHANES database with the NDI database, with follow-up until 31 December 2019, constructing a longitudinal survival cohort. Our research findings suggest that among patients with CKM syndrome, there is a linear positive association between NHR and CVD mortality. With the increase in NHR, the risk of CVD mortality increased by up to 2.5 times at most. In the subgroup analysis, the relationship between NHR and the risk of CVD mortality was consistent across different subgroups. In conclusion, this study provides new evidence for the diagnosis and long-term prognosis of CKM syndrome.

As a newly emerged medical concept in recent years, CKM syndrome is a highly complex and multifaceted disorder that exerts a profound impact on the overall health status and quality of life of patients. In this study, the definition criteria for CKM syndrome focused on stages 3 to 4. Specifically, individuals at stage 3 are characterized by a high risk of developing CKD or CVD, while patients at stage 4 have been clearly diagnosed with CVD. Currently, no research has explored the relationship between NHR and CKM syndrome. However, some studies have shown that a one-unit increase in NHR is associated with a 9% increase in the prevalence of CKD (OR: 1.09, 95% confidence interval: 1.06–1.11, *p* < 0.0001) (10). Other research has indicated a positive association between elevated NHR levels and the prevalence of Metabolic Dysfunction-Associated Steatotic Liver Disease (MASLD) (22, 23). The study by Gao et al. (9) demonstrated that among American adults, a one-thousandth unit increase in SII is associated with approximately 48% increase in the prevalence of CKM syndrome, suggesting that a higher level of inflammation is a strong risk factor for CKM syndrome. This study has expanded my knowledge of this field. It was found that per SD increase in NHR is associated with a 25% increase in the prevalence of CKM syndrome, emphasizing that NHR may serve as a new diagnostic tool for CKM syndrome. In addition, previous studies have also shown that NHR can significantly predict the CVD mortality in the general American population (11). Our study reveals that NHR is a powerful predictor of cardiovascular outcomes in patients with CKM syndrome.

The pathophysiological mechanism of CKM syndrome presents a complex interactive network, with its core being the multidirectional associations among metabolic risk factors, CKD, and the cardiovascular system. When analyzing the root causes of the onset of CKM syndrome, common initiating factors include excessive accumulation or dysfunction of adipose tissue or both. Among them, the dysfunctional visceral adipose tissue is particularly harmful as it can secrete bioactive substances with pro-inflammatory and pro-oxidative properties, which can cause damage to important tissues such as arteries, heart, and kidneys (24–26). Once the inflammatory response is initiated, the body’s sensitivity to insulin decreases, which in turn leads to abnormal glucose tolerance (25, 27). The development of MASLD will exacerbate the systemic inflammatory response and insulin resistance (28). More seriously, MASLD not only has systemic effects but also becomes the primary cause of liver failure, resulting in liver transplantation (29). When pro-oxidative and pro-inflammatory mediators are released into the systemic circulation, a series of chain reactions will be triggered. In terms of atherosclerosis and myocardial injury, it will cause damage to the vascular endothelium and exacerbate the dysfunction of myocardial cells. The processes of glomerulosclerosis, tubular inflammation, and renal fibrosis will accelerate the pathological changes in renal tissues. From the perspective of metabolic risk factors, it promotes their further deterioration. In addition to the systemic effects of adipose tissue, ectopic fat is also a potential risk factor. It can serve as a source of local inflammatory mediators, causing compressive organ damage. When ectopic fat accumulates in the epicardium and pericardium, it is likely to induce arrhythmia, myocardial dysfunction, and coronary atherosclerosis. If it accumulates in and around the kidneys, it will lead to hypertension and abnormal fluctuations in blood pressure (27, 30). NHR reveals the internal connection between the two pathways of lipid metabolism and the inflammatory state, reflecting the interaction between them (31). Neutrophils, as inflammatory markers, also have pro-oxidative functions (32). HDL-C plays a key role in the reverse cholesterol transport in the human body and provides protection with its anti-inflammatory, anti-oxidative, and antithrombotic properties (33, 34). However, in the “microenvironment” within the body, neutrophils can weaken the antioxidant and anti-inflammatory effects of HDL-C and promote the oxidation of LDL-C through degranulation (35, 36). When the level of HDL-C decreases, the antioxidant capacity declines and oxidative stress increases. As one of the key pathogenic mechanisms of CKM syndrome, oxidative stress can damage vascular endothelial cells, trigger an inflammatory response, and thus affect the functions of the cardiovascular and renal systems, facilitating the development of CKM syndrome. Overall, it is logical that NHR, which reflects the relationship between neutrophils and HDL-C, has a linear positive association with CKM syndrome.

In our longitudinal cohort study, during an average follow-up period of 6.95 years, NHR significantly predicted the risk of CVD mortality in patients with CKM syndrome. This indicates that NHR not only serves as a diagnostic indicator for CKM syndrome but may also mediate the deterioration of cardiovascular function in this disease. In patients with CKM syndrome, persistent metabolic disorders lead to dysfunction of adipose tissue, which may result in the aggregation and activation of a large number of neutrophils (37). On one hand, these neutrophils release inflammatory factors to exacerbate the inflammatory response of the body and damage the vascular endothelium. On the other hand, they also interfere with the normal function of HDL-C, weakening its protective properties such as antioxidation, anti-inflammation, and antithrombosis and promoting the oxidation of low-density lipoprotein cholesterol (LDL-C), accelerating the process of atherosclerosis (35, 36, 38). Over time, during the average 6.95-year follow-up, the cumulative effect of this imbalance between inflammation and lipid metabolism becomes more and more pronounced, directly reflected by an increase in NHR. Moreover, the elevated NHR further indicates the aggravation of damage to the cardiovascular system, leading to a sharp increase in the risk of CVD mortality.

## Advantages and limitations

Our study has the following advantages. First, the study has a large sample size, and by taking into account the design weights of NHANES, it is able to represent the entire population of the United States. Second, this study simultaneously explored the relationship between NHR and CKM syndrome, as well as its associated mortality risk. Third, the results of our study are relatively robust, indicating a strong association between NHR and CKM syndrome and its mortality risk among different populations. In conclusion, this study provides strong evidence for identifying the new concept of CKM syndrome.

It should be noted that our study inevitably has some limitations. First, considering that the association between CKM syndrome and NHR in the cross-sectional study may have a causal direction, it is necessary to investigate the relationship between the two in a longitudinal cohort in the future. Second, our study may have overlooked some variables that simultaneously affect both NHR and CKM syndrome, which may lead to a bias in the estimated strength of the association between them. Third, the calculation of NHR is based on data from a single measurement. Therefore, it may be affected by the subject’s stress state at that time, resulting in bias. Fourth, this study may have underestimated the prevalence of CKM syndrome. As Aggarwal et al. (3) pointed out, in the NHANES database, the lack of evidence from clinical examinations such as echocardiography may lead to an underestimation of the prevalence of advanced CKM syndrome.

## Conclusion

In conclusion, this study has revealed the associations between NHR and CKM syndrome and its prognosis. NHR can serve as a potential biomarker for CKM syndrome and may be used to predict the cardiovascular outcomes of CKM syndrome.

## Data Availability

Publicly available datasets were analyzed in this study. This data can be found here: The website for cross-sectional data is https://wwwn.cdc.gov/nchs/nhanes/; the website for survival data is https://www.cdc.gov/nchs/ndi/?CDC_AAref_Val=https://www.cdc.gov/nchs/ndi/index.htm.

## Glossary

AHA: American Heart Association
BMI: Body mass index
CI: Confidence interval
CKD: Chronic kidney disease
CKM: Cardiovascular-kidney-metabolic syndrome
CVD: Cardiovascular diseases
DM: Diabetes mellitus
GFR: Glomerular filtration rate
HDL-C: High-density lipoprotein cholesterol
HR: Hazard ratio
ICD-10: The 10th revision of the International Statistical Classification of Diseases
KDIGO: Kidney Disease Improving Global Outcomes
LDL-C: Low-density lipoprotein cholesterol
MASLD: Metabolic Dysfunction-Associated Steatotic Liver Disease
NDI: National Death Index
NHANES: National Health and Nutrition Examination Survey
NHR: Neutrophil/high-density lipoprotein cholesterol ratio
OR: Odds ratio
PIR: Poverty income ratio
PREVENT: Predicting Risk of CVD EVENTs
RCS: Restricted cubic spline
SII: Systemic immune-inflammation index
T2DM: Type 2 diabetes mellitus
TC: Total cholesterol
VIF: Variance inflation factor

## References

[1] Ndumele CE Neeland IJ Tuttle KR Chow SL Mathew RO Khan SS. A synopsis of the evidence for the science and clinical management of cardiovascular-kidney-metabolic (CKM) syndrome: a scientific statement from the American Heart Association. Circulation. (2023) 148:1636–64. doi: 10.1161/cir.0000000000001186, PMID: 37807920

[2] Sebastian SA Padda I Johal G. Cardiovascular-kidney-metabolic (CKM) syndrome: a state-of-the-art review. Curr Probl Cardiol. (2024) 49:102344. doi: 10.1016/j.cpcardiol.2023.102344, PMID: 38103820

[3] Aggarwal R Ostrominski JW Vaduganathan M. Prevalence of cardiovascular-kidney-metabolic syndrome stages in US adults, 2011–2020. JAMA. (2024) 331:1858–60. doi: 10.1001/jama.2024.6892, PMID: 38717747 PMC11079779

[4] Weisberg SP McCann D Desai M Rosenbaum M Leibel RL Ferrante AW Jr. Obesity is associated with macrophage accumulation in adipose tissue. J Clin Invest. (2003) 112:1796–808. doi: 10.1172/jci19246, PMID: 14679176 PMC296995

[5] Ferrucci L Fabbri E. Inflammageing: chronic inflammation in ageing, cardiovascular disease, and frailty. Nat Rev Cardiol. (2018) 15:505–22. doi: 10.1038/s41569-018-0064-2, PMID: 30065258 PMC6146930

[6] Attiq A Afzal S Ahmad W Kandeel M. Hegemony of inflammation in atherosclerosis and coronary artery disease. Eur J Pharmacol. (2024) 966:176338. doi: 10.1016/j.ejphar.2024.176338, PMID: 38242225

[7] Zoja C Abbate M Remuzzi G. Progression of renal injury toward interstitial inflammation and glomerular sclerosis is dependent on abnormal protein filtration. Nephrol Dial Transplant. (2015) 30:706–12. doi: 10.1093/ndt/gfu261, PMID: 25087196

[8] Sharma S Smyth B. From proteinuria to fibrosis: an update on pathophysiology and treatment options. Kidney Blood Press Res. (2021) 46:411–20. doi: 10.1159/000516911, PMID: 34130301

[9] Gao C Gao S Zhao R Shen P Zhu X Yang Y. Association between systemic immune-inflammation index and cardiovascular-kidney-metabolic syndrome. Sci Rep. (2024) 14:19151. doi: 10.1038/s41598-024-69819-0, PMID: 39160192 PMC11333479

[10] Li X Cui L Xu H. Association between systemic inflammation response index and chronic kidney disease: a population-based study. Front Endocrinol. (2024) 15:1329256. doi: 10.3389/fendo.2024.1329256, PMID: 38455650 PMC10917959

[11] Jiang M Sun J Zou H Li M Su Z Sun W. Prognostic role of neutrophil to high-density lipoprotein cholesterol ratio for all-cause and cardiovascular mortality in the general population. Front Cardiovasc Med. (2022) 9:807339. doi: 10.3389/fcvm.2022.807339, PMID: 35211525 PMC8861276

[12] Chen T Chen H Xiao H Tang H Xiang Z Wang X. Comparison of the value of neutrophil to high-density lipoprotein cholesterol ratio and lymphocyte to high-density lipoprotein cholesterol ratio for predicting metabolic syndrome among a population in the southern coast of China. Diabetes Metab Syndr Obes. (2020) 13:597–605. doi: 10.2147/dmso.S238990, PMID: 32184639 PMC7053653

[13] Ndumele CE Rangaswami J Chow SL Neeland IJ Tuttle KR Khan SS. Cardiovascular-kidney-metabolic health: a presidential advisory from the American Heart Association. Circulation. (2023) 148:1606–35. doi: 10.1161/CIR.0000000000001184, PMID: 37807924

[14] ZhuRWangRHeJWangLChenHWangY. Associations of cardiovascular-kidney-metabolic syndrome stages with premature mortality and the role of social determinants of health. J Nutr Health Aging. (2025) 29:100504. doi: 10.1016/j.jnha.2025.100504, PMID: 39952015

[15] Stevens PE Ahmed SB Carrero JJ Foster B Francis A Hall RK. KDIGO 2024 clinical practice guideline for the evaluation and management of chronic kidney disease. Kidney Int. (2024) 105:S117–314. doi: 10.1016/j.kint.2023.10.018, PMID: 38490803

[16] Khan SS Matsushita K Sang Y Ballew SH Grams ME Surapaneni A. Development and validation of the American Heart Association’s PREVENT equations. Circulation. (2024) 149:430–49. doi: 10.1161/circulationaha.123.067626, PMID: 37947085 PMC10910659

[17] Wu L Huang Z. Elevated triglyceride glucose index is associated with advanced cardiovascular kidney metabolic syndrome. Sci Rep. (2024) 14:31352. doi: 10.1038/s41598-024-82881-y, PMID: 39732891 PMC11682451

[18] Wen X Wang M Xu X Li T. Exposure to per- and polyfluoroalkyl substances and mortality in U.S. adults: a population-based cohort study. Environ Health Perspect. (2022) 130:67007. doi: 10.1289/ehp10393 35731224 PMC9215707

[19] Hou XZ Lv YF Li YS Wu Q Lv QY Yang YT. Association between different insulin resistance surrogates and all-cause mortality in patients with coronary heart disease and hypertension: NHANES longitudinal cohort study. Cardiovasc Diabetol. (2024) 23:86. doi: 10.1186/s12933-024-02173-7, PMID: 38419039 PMC10903030

[20] Du YZ Yang JQ Yao JM Zhang CT Liu YF. Association between the neutrophil-to-high-density lipoprotein cholesterol ratio with kidney stone risk: a cross-sectional study. Front Endocrinol. (2025) 16:1523890. doi: 10.3389/fendo.2025.1523890, PMID: 39963279 PMC11830614

[21] Zhang J. Associations of neutrophil/high-density lipoprotein cholesterol ratio with frailty and its mortality. Front Endocrinol. (2024) 15:1495139. doi: 10.3389/fendo.2024.1495139 PMC11743577 39835260

[22] Lu Y Xu X Wu J Ji L Huang H Chen M. Association between neutrophil-to-high-density lipoprotein cholesterol ratio and metabolic dysfunction-associated steatotic liver disease and liver fibrosis in the US population: a nationally representative cross-sectional study using NHANES data from 2017 to 2020. BMC Gastroenterol. (2024) 24:300. doi: 10.1186/s12876-024-03394-6, PMID: 39237899 PMC11378436

[23] Zhu N Li Y Lin Y Cui X Li X. Association between neutrophil-to-high-density lipoprotein cholesterol ratio and non-alcoholic fatty liver disease or metabolic dysfunction-associated steatotic liver disease: evidence from NHANES 2017–2020. Front Med. (2024) 11:1491858. doi: 10.3389/fmed.2024.1491858, PMID: 39882525 PMC11774988

[24] Powell-Wiley TM Poirier P Burke LE Després JP Gordon-Larsen P Lavie CJ. Obesity and cardiovascular disease: a scientific statement from the American Heart Association. Circulation. (2021) 143:e984–e1010. doi: 10.1161/cir.0000000000000973, PMID: 33882682 PMC8493650

[25] Rana MN Neeland IJ. Adipose tissue inflammation and cardiovascular disease: an update. Curr Diab Rep. (2022) 22:27–37. doi: 10.1007/s11892-021-01446-9, PMID: 35179694

[26] Després JP Carpentier AC Tchernof A Neeland IJ Poirier P. Management of obesity in cardiovascular practice: JACC focus seminar. J Am Coll Cardiol. (2021) 78:513–31. doi: 10.1016/j.jacc.2021.05.035, PMID: 34325840 PMC8609918

[27] Yano Y Vongpatanasin W Ayers C Turer A Chandra A Carnethon MR. Regional fat distribution and blood pressure level and variability: the Dallas heart study. Hypertension. (2016) 68:576–83. doi: 10.1161/hypertensionaha.116.07876, PMID: 27432862 PMC4982814

[28] Rinella ME Lazarus JV Ratziu V Francque SM Sanyal AJ Kanwal F. A multisociety delphi consensus statement on new fatty liver disease nomenclature. Hepatology. (2023) 6:78. doi: 10.1097/HEP.0000000000000520 PMC10653297 37363821

[29] Wong RJ Singal AK. Trends in liver disease etiology among adults awaiting liver transplantation in the United States, 2014–2019. JAMA Netw Open. (2020) 3:e1920294. doi: 10.1001/jamanetworkopen.2019.20294, PMID: 32022875 PMC12124732

[30] Sheng X Qiu C Liu H Gluck C Hsu JY He J. Systematic integrated analysis of genetic and epigenetic variation in diabetic kidney disease. Proc Natl Acad Sci USA. (2020) 117:29013–24. doi: 10.1073/pnas.2005905117, PMID: 33144501 PMC7682409

[31] Qing G Bao C Yang Y Wei B. Association between neutrophil to high-density lipoprotein cholesterol ratio (NHR) and depression symptoms among the United States adults: a cross-sectional study. Lipids Health Dis. (2024) 23:215. doi: 10.1186/s12944-024-02204-y, PMID: 39003458 PMC11245866

[32] Stojkov D Gigon L Peng S Lukowski R Ruth P Karaulov A. Physiological and pathophysiological roles of metabolic pathways for NET formation and other neutrophil functions. Front Immunol. (2022) 13:826515. doi: 10.3389/fimmu.2022.826515, PMID: 35251008 PMC8889909

[33] Wen SY Tamilselvi S Shen CY Day CH Chun LC Cheng LY. Protective effect of HDL on NADPH oxidase-derived super oxide anion mediates hypoxia-induced cardiomyocyte apoptosis. PLoS One. (2017) 12:e0179492. doi: 10.1371/journal.pone.0179492, PMID: 28617849 PMC5472312

[34] Soran H Schofield JD Durrington PN. Antioxidant properties of HDL. Front Pharmacol. (2015) 6:222. doi: 10.3389/fphar.2015.00222 26528181 PMC4607861

[35] Chiesa ST Charakida M. High-density lipoprotein function and dysfunction in health and disease. Cardiovasc Drugs Ther. (2019) 33:207–19. doi: 10.1007/s10557-018-06846-w, PMID: 30675710 PMC6509080

[36] Tucker B Ephraums J King TW Abburi K Rye KA Cochran BJ. Impact of impaired cholesterol homeostasis on neutrophils in atherosclerosis. Arterioscler Thromb Vasc Biol. (2023) 43:618–27. doi: 10.1161/atvbaha.123.316246, PMID: 36951066

[37] Scalia R. The microcirculation in adipose tissue inflammation. Rev Endocr Metab Disord. (2013) 14:69–76. doi: 10.1007/s11154-013-9236-x, PMID: 23378133 PMC6438368

[38] Kattoor AJ Pothineni NVK Palagiri D Mehta JL. Oxidative stress in atherosclerosis. Curr Atheroscler Rep. (2017) 19:42. doi: 10.1007/s11883-017-0678-6, PMID: 28921056

