# Supplementary material for: Association of neutrophil/high-density lipoprotein cholesterol ratio with the cardiovascular-kidney-metabolic syndrome and its cardiovascular mortality
Source: Front Nutr. 2025 Jun 4;12:1594041. doi: 10.3389/fnut.2025.1594041 (PMC12173847; doi:10.3389/fnut.2025.1594041)
Supplement: Supplementary file 1 [file Table_1.DOCX]

**Association of neutrophil/high-density lipoprotein cholesterol ratio with the cardiovascular-kidney-metabolic syndrome and its cardiovascular mortality**

Yaying Xu^1^*

^1^ Department of Endocrinology, The First Affiliated Hospital, and College of Clinical Medicine of Henan University of Science and Technology, Luoyang, China.

* Correspondence: Yaying Xu

Email: [xyy2015haust@126.com](mailto:xyy2015haust@126.com)

## Supplementaty Table 1: Definition of cardiovascular-kidney-metabolic syndrome stages:

| Cardiovascular-kidney-metabolic (CKM) syndrome stages were classified using data from NHANES 2017-2018, according to the 2023 AHA Presidential Advisory on CKM Health ([1](#_ENREF_1)). Definitions were adapted based on available NHANES data: | |  |
| --- | --- | --- |
|  |  |  |
| CKM Stage 0: | Participants with normal body mass index (BMI, <25 kg/m²), waist circumference (<88 cm for women, <102 cm for men), normoglycemia (fasting blood glucose [FBG] <100 mg/dL, glycated hemoglobin [HbA1c] <5.7%), normotension (systolic blood pressure [BP] <130 mmHg, diastolic BP <80 mmHg), normal lipid profile (triglycerides <135 mg/dL), and no evidence of chronic kidney disease (CKD) or clinical/subclinical cardiovascular disease (CVD). |  |
|  |  |  |
|  |  |  |
|  |  |  |
| CKM Stage 1: | Participants with elevated BMI (≥25 kg/m²), increased waist circumference (≥88 cm for women, ≥102 cm for men), or prediabetes (HbA1c 5.7%-6.4% or FBG 100-125 mg/dL), without the presence of other metabolic risk factors or CKD. |  |
|  |  |  |
|  |  |  |
|  |  |  |
| CKM Stage 2: | Participants with metabolic risk factors or moderate-to-high-risk CKD per KDIGO guidelines ([2](#_ENREF_2)). Metabolic risk factors included elevated triglycerides (≥135 mg/dL), hypertension, diabetes, or metabolic syndrome (≥3 of the following: elevated waist circumference, low HDL [<40 mg/dL for men, <50 mg/dL for women], elevated triglycerides [≥150 mg/dL], elevated BP [systolic ≥130 mmHg, diastolic ≥80 mmHg], or prediabetes). |  |
|  |  |  |
|  |  |  |
|  |  |  |
| CKM Stage 3: | Participants with very-high-risk CKD (KDIGO criteria) or high 10-year CVD risk (≥20%) based on the AHA PREVENT equations [3]. High risk was defined as ≥20% 10- year CVD risk (based on recommended thresholds [https://professional.heart.org/en/guidelines-and-statements/prevent-calculator]). Very high-risk CKD was characterized by either Stage G4 or G5 CKD (GFR < 30 mL/min/1.73 m²) or a classification of very high risk based on KDIGO guidelines, determined by GFR and urinary albumin-to-creatinine ratio ([2](#_ENREF_2)). |  |
|  |  |  |
|  |  |  |
|  |  |  |
| CKM Stage 4: | Participants with self-reported established CVD, including coronary heart disease, angina, myocardial infarction, heart failure, and stroke. Atrial fibrillation and peripheral artery disease were not included due to data unavailability. |  |
|  |  |  |
|  |  |  |
|  |  |  |

1. Ndumele CE, Neeland IJ, Tuttle KR, Chow SL, Mathew RO, Khan SS, et al. A Synopsis of the Evidence for the Science and Clinical Management of Cardiovascular-Kidney-Metabolic (CKM) Syndrome: A Scientific Statement From the American Heart Association. Circulation. 2023;148(20):1636-64.

2. KDIGO 2021 Clinical Practice Guideline for the Management of Glomerular Diseases. Kidney Int. 2021;100(4s):S1-s276.
